# Supplementary material for: Low-power WALTZ decoupling under magic-angle spinning NMR
Source: Magn Reson (Gott). 2024 Nov 19;5(2):153–66. doi: 10.5194/mr-5-153-2024 (PMC12178133; doi:10.5194/mr-5-153-2024)
Supplement: The supplement related to this article is available online at: https://doi.org/10.5194/mr-5-153-2024-supplement. [file mr-5-153-2024-supplement.pdf]

Supplement of Magn. Reson., 5, 153–166, 2024
https://doi.org/10.5194/mr-5-153-2024-supplement
© Author(s) 2024. CC BY 4.0 License.

Open Access
MAGNETIC RESONANCE

Supplement of

Low-power WALTZ decoupling under magic-angle spinning NMR

Luzian Thomas and Matthias Ernst

Correspondence to: Matthias Ernst (maer@ethz.ch)

The copyright of individual parts of the supplement might differ from the article licence.

Contents

S1 Parameters used for numerical simulations **1**
S1.1 Two-spin system . . . 1
S1.2 Three-spin system . . . 1

S2 Equivalent plots for WALTZ-16 decoupling **1**

S3 Additional figures **10**

S1 Parameters used for numerical simulations

Numerical simulations were carried out for two-spin and three-spin systems with the following parameters that correspond to values obtained using a DFT simulation of the CH_2 group in glycine. The time-dependent Hamiltonian was approximated by time slicing the rotor period in a variable number of steps such that the basic 90° pulses of the WALTZ sequence would fit into the raster of the time slices. The spinning frequency was always set to 100 kHz and the rf-field amplitude calculated from the pulse length. Powder averaging over 1054 distinct (α, β, γ) orientations was achieved by using the Zaremba-Conroy-Wolfsberg (ZCW) scheme (V.B. Cheng, H.H. Suzukawa, M. Wolfsberg, Investigations of a Nonrandom Numerical-Method for Multidimensional Integration, J. Chem. Phys. 59 (1973) 3992–3999).

S1.1 Two-spin system

All chemical shifts were set to zero (on-resonance irradiation) unless stated otherwise. The heteronuclear dipolar coupling was set to $\delta_{\mathrm{I_1S}}/(2\pi)$ = -45.303 kHz with Euler angles $(\alpha_{\mathrm{I_1S}}, \beta_{\mathrm{I_1S}}, \gamma_{\mathrm{I_1S}}) = (0°, -81.6°, -137.3°)$. If included, the J_{IS} coupling was set to $J_{\mathrm{I_1S}}$=150 Hz and the CSA tensor of the proton to $\delta_{\mathrm{I_1}}/(2\pi)$= 1.53 kHz, $\eta_{\mathrm{I_1}}/(2\pi)$= 0.51 and Euler angles $(\alpha_{\mathrm{I_1}}, \beta_{\mathrm{I_1}}, \gamma_{\mathrm{I_1}}) = (-86.9°, 81.1°, -54.3°)$.

S1.2 Three-spin system

In the three-spin simulations, the additional spin system parameters are: $\delta_{\mathrm{I_2S}}/(2\pi)$ = -45.220 kHz with Euler angles $(\alpha_{\mathrm{I_2S}}, \beta_{\mathrm{I_2S}}, \gamma_{\mathrm{I_2S}}) = (0°, -86.7°, -30.1°)$; $J_{\mathrm{I_2S}}$=150 Hz; $\delta_{\mathrm{I_2}}/(2\pi)$= 1.61 kHz, $\eta_{\mathrm{I_2}}/(2\pi)$= 0.64 and Euler angles $(\alpha_{\mathrm{I_2}}, \beta_{\mathrm{I_2}}, \gamma_{\mathrm{I_2}}) = (91.7°, 86.4°, 50.2°)$; $\delta_{\mathrm{I_1I_2}}/(2\pi)$ = -43.770 kHz with Euler angles $(\alpha_{\mathrm{I_1I_2}}, \beta_{\mathrm{I_1I_2}}, \gamma_{\mathrm{I_1I_2}}) = (0°, -86.9°, -173.9°)$. For some of the simulations (as indicated in the text), the magnitude of the homonuclear dipolar coupling was set to zero.

S2 Equivalent plots for WALTZ-16 decoupling

In this section we collect all the plots for WALTZ-16 decoupling that are equivalent to the WALTZ-64 decoupling shown in the main manuscript. All simulation and experimental parameters are the same as in the WALTZ-64 figures.

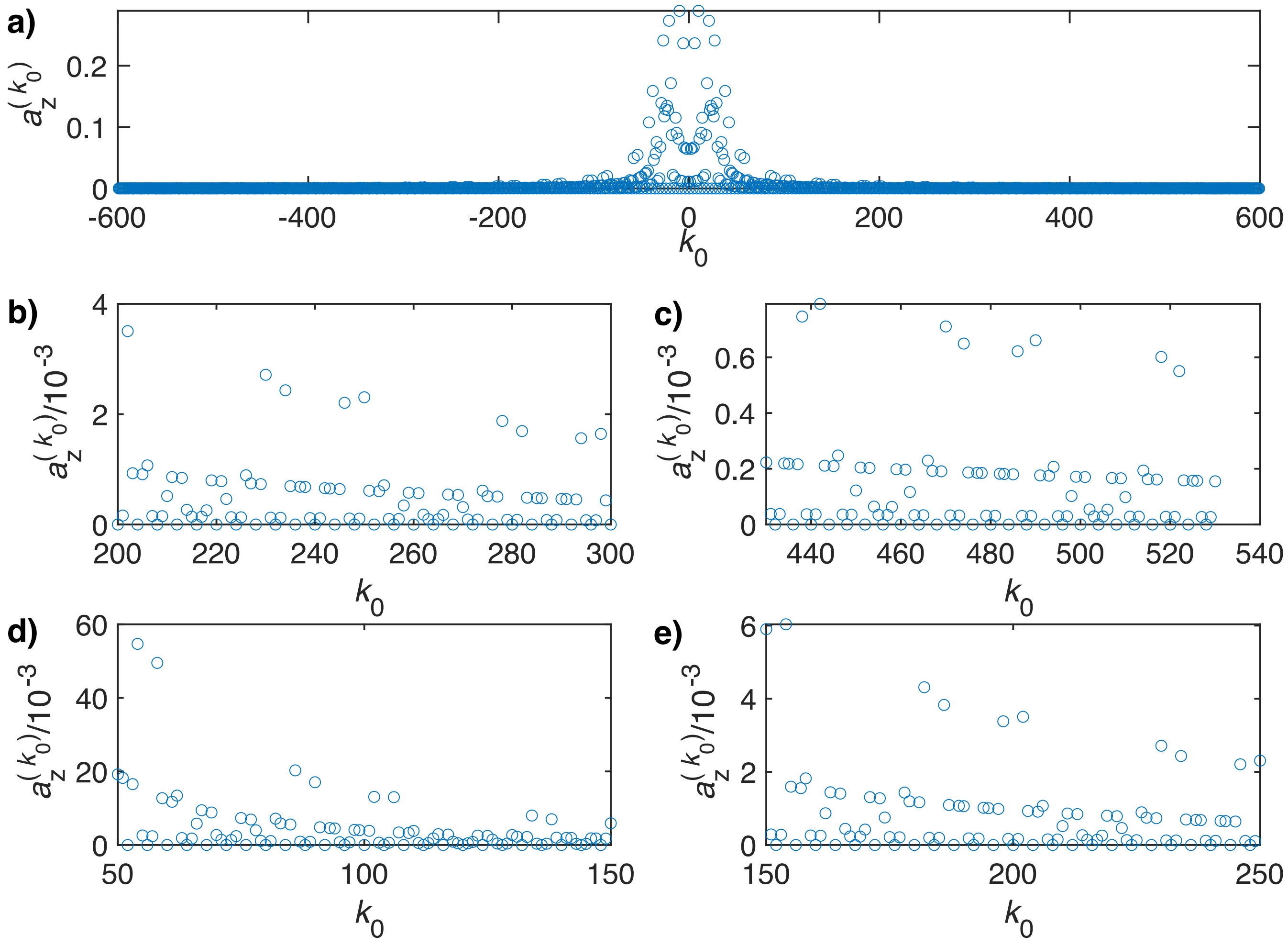

Figure S1. Plot of the Fourier coefficients $a_z^{(k_0)}$ as a function of k_0 for the WALTZ-16 pulse sequence assuming ideal rectangular pulses. One can clearly see that even for very high values of k_0 many of the resonance condition still have a significant contribution. a) Complete range from $k_0 = -600$ to 600. b) Enlarged range around $k_0 = 240$ corresponding to a ratio of $\omega_\mathrm{r}/\omega_1 = 10$ for $n_0 = 1$. c) Enlarged range around $k_0 = 480$ corresponding to a ratio of $\omega_\mathrm{r}/\omega_1 = 10$ for $n_0 = 2$. d) Enlarged range around $k_0 = 96$ corresponding to a ratio of $\omega_\mathrm{r}/\omega_1 = 4$ for $n_0 = 1$. c) Enlarged range around $k_0 = 192$ corresponding to a ratio of $\omega_\mathrm{r}/\omega_1 = 4$ for $n_0 = 2$.

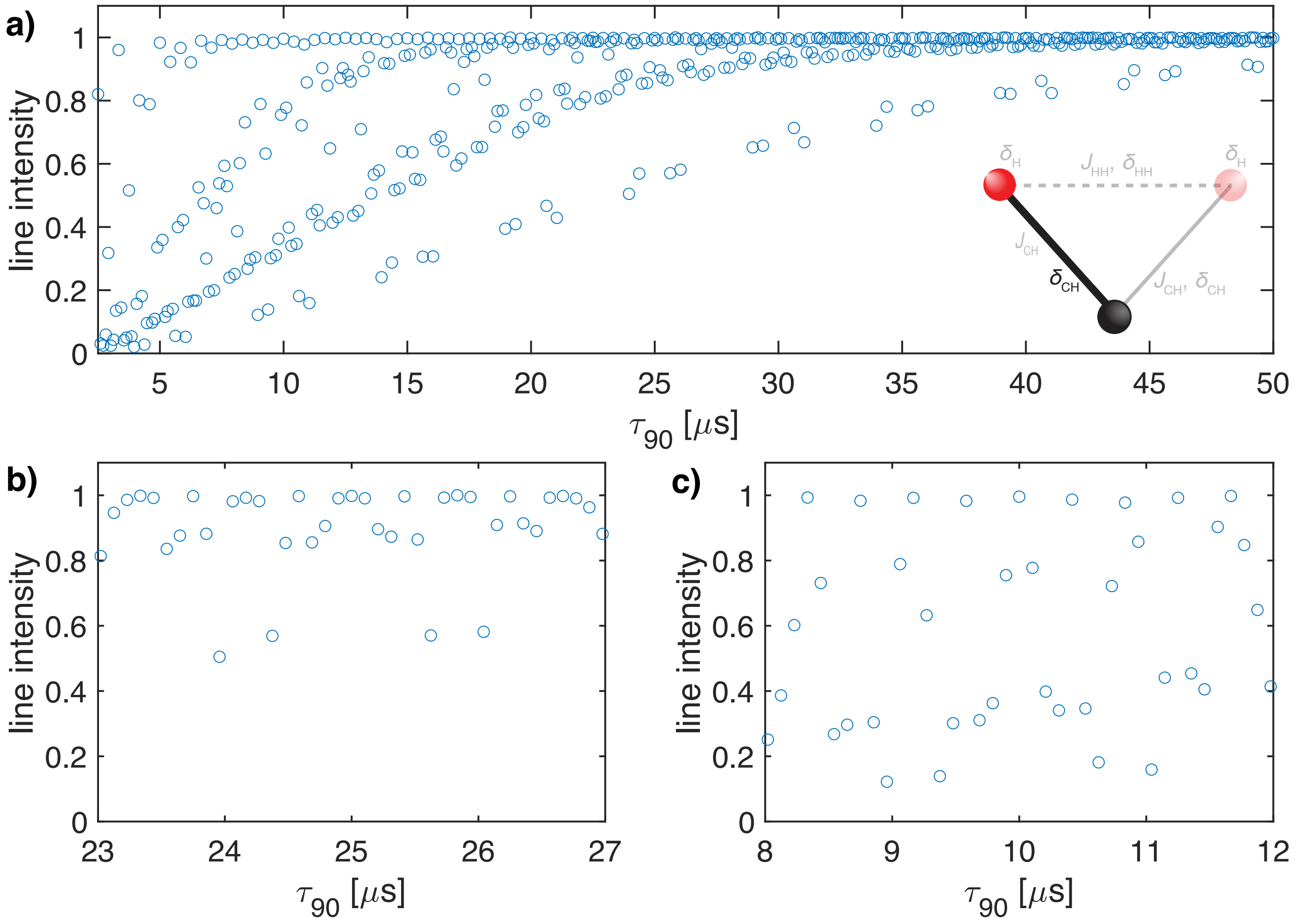

Figure S2. Plot of the simulated line intensity in a two-spin system with only the heteronuclear dipolar coupling as a function of the pulse length τ_{90} for the WALTZ-16 pulse sequence assuming ideal rectangular pulses. The corresponding rf-field amplitude for each value of τ_{90} can be calculated as $\nu_1 = 1/(4\tau_{90})$. The pulse length correspond to values of $\tau_{90} = k_0\tau_r/(4 * z_0)$. a) Complete range from τ_{90} = 2.5 to 50 µs corresponding to k_0 = 24 to 480. b) Enlarged range around τ_{90} = 25 µs corresponding to an ideal B_1 field of 10 kHz. c) Enlarged range around τ_{90} = 10 µs corresponding to an ideal B_1 field of 25 kHz. As described in the materials and methods section, all simulations are processed with an exponential apodization of 30 Hz with an intensity of one corresponding to a non decaying line.

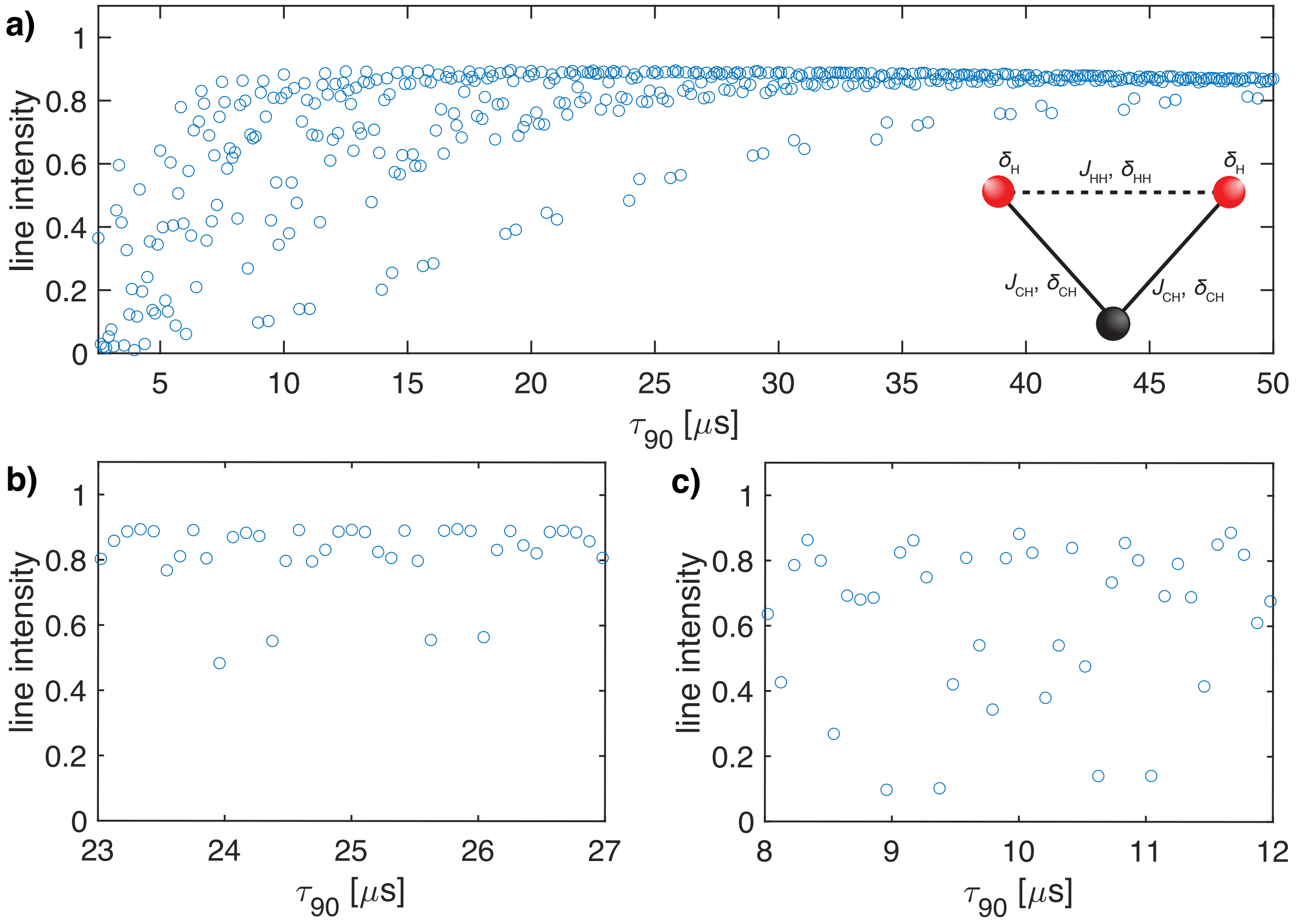

Figure S3. Plot of the simulated line intensity in a CH_2 three-spin system with dipolar couplings and CSA tensors as a function of the pulse length τ_{90} for the WALTZ-16 pulse sequence assuming ideal rectangular pulses. The corresponding rf-field amplitude for each value of τ_{90} can be calculated as $\nu_1 = 1/(4\tau_{90})$. The pulse length correspond to values of $\tau_{90} = k_0\tau_r/(4 * z_0)$ a) Complete range from τ_{90} = 2.5 to 50 µs corresponding to k_0 = 24 to 480. b) Enlarged range around τ_{90} = 25 µs corresponding to an ideal B_1 field of 10 kHz. c) Enlarged range around τ_{90} = 10 µs corresponding to an ideal B_1 field of 25 kHz. As described in the materials and methods section, all simulations are processed with an exponential apodization of 30 Hz with an intensity of one corresponding to a non decaying line.

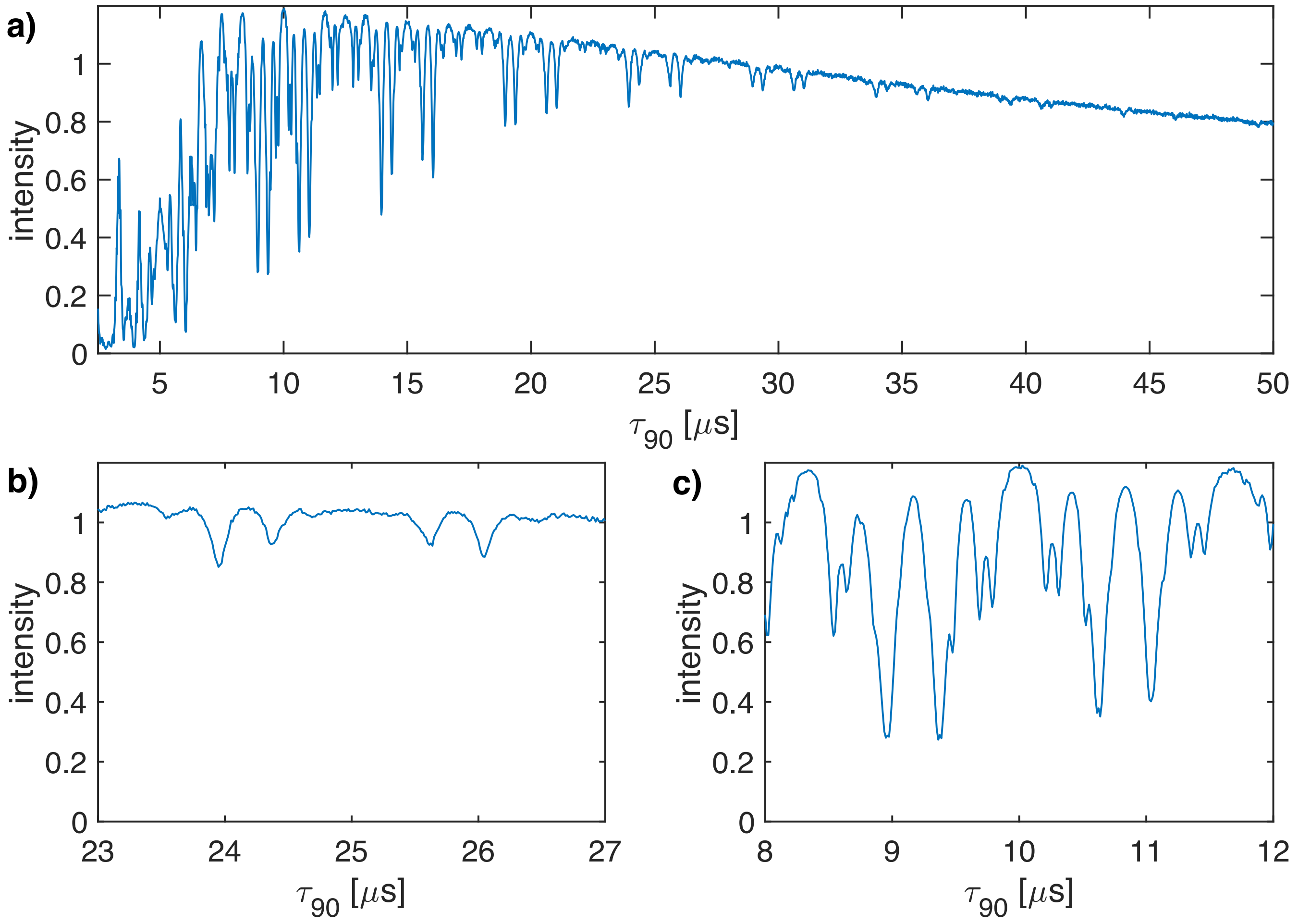

Figure S4. Plot of the measured line intensity of the CH_2 group in 1,2-^{13}C glycine ethyl ester as a function of the pulse length τ_{90} for the WALTZ-16 pulse sequence at 100 kHz MAS. The rf-field amplitude was adjusted such that the flip angle was always 90°. The increment of the pulse length was set to 12.5 ns. a) Complete range from τ_{90} = 2.5 to 50 µs. b) Enlarged range around τ_{90} = 25 µs corresponding to a B_1 field around 10 kHz. c) Enlarged range around τ_{90} = 10 µs corresponding to a B_1 field around 25 kHz. While the numerical calculations and simulations of Figs. S1-S3 could be carried out exactly on the resonance conditions, this was not possible for the experimental data due to the limited time resolution of the pulse programmer and the limited stability of the MAS rotation. Therefore, the experimental data are displayed as a line plot with the highest possible time resolution.

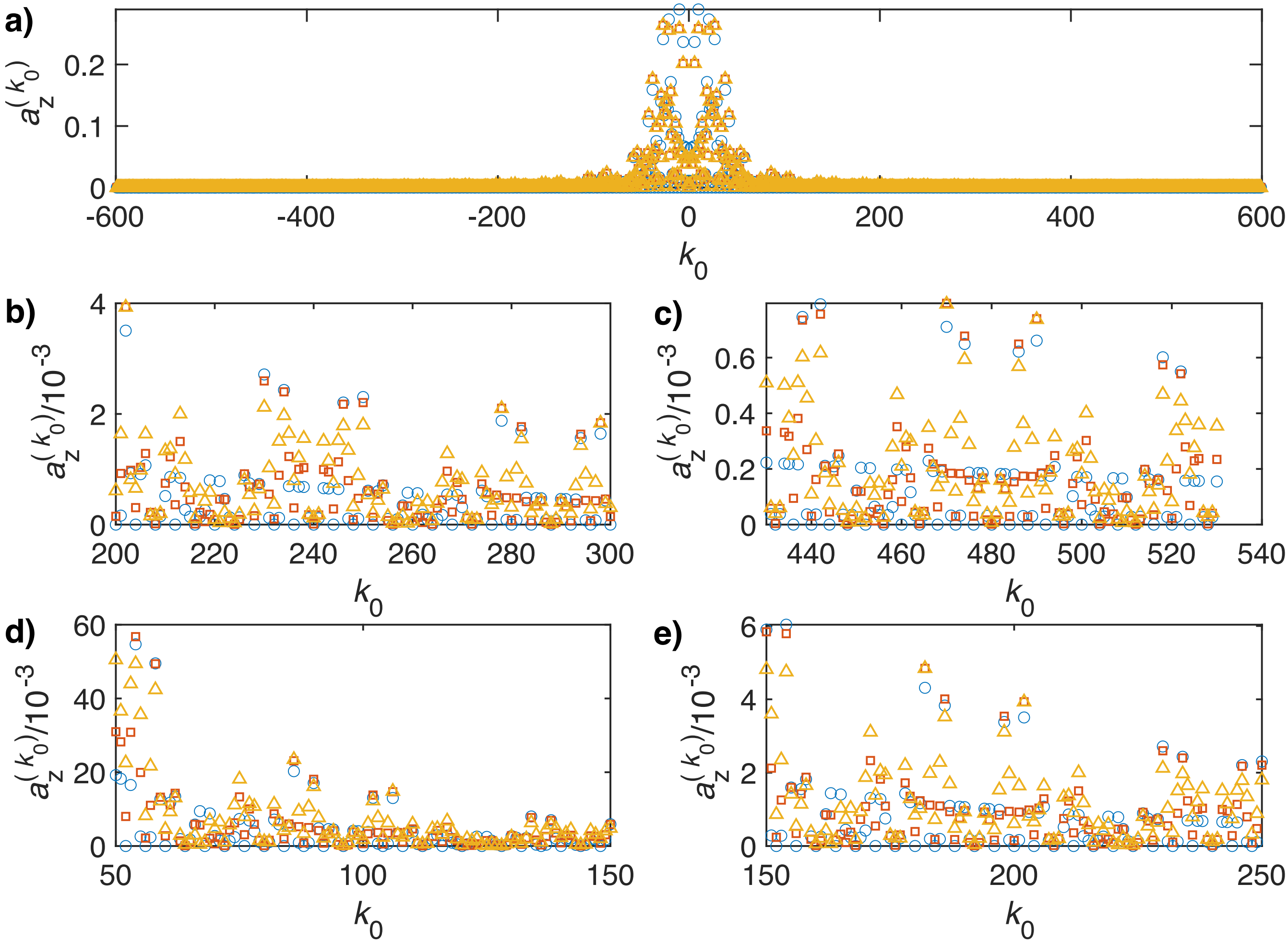

Figure S5. Plot of the Fourier coefficients $a_z^{(k_0)}$ as a function of k_0 for the WALTZ-16 pulse sequence assuming ideal rectangular pulses. Blue circles correspond to the ideal B_1 field, red squares to a B_1 field that is increased by 10% and yellow triangles to one that is increased by 20%. a) Complete range from $k_0 = -600$ to 600. b) Enlarged range around $k_0 = 240$ corresponding to a ratio of $\omega_\mathrm{r}/\omega_1 = 10$ for $n_0 = 1$. c) Enlarged range around $k_0 = 480$ corresponding to a ratio of $\omega_\mathrm{r}/\omega_1 = 10$ for $n_0 = 2$. d) Enlarged range around $k_0 = 96$ corresponding to a ratio of $\omega_\mathrm{r}/\omega_1 = 4$ for $n_0 = 1$. c) Enlarged range around $k_0 = 192$ corresponding to a ratio of $\omega_\mathrm{r}/\omega_1 = 4$ for $n_0 = 2$.

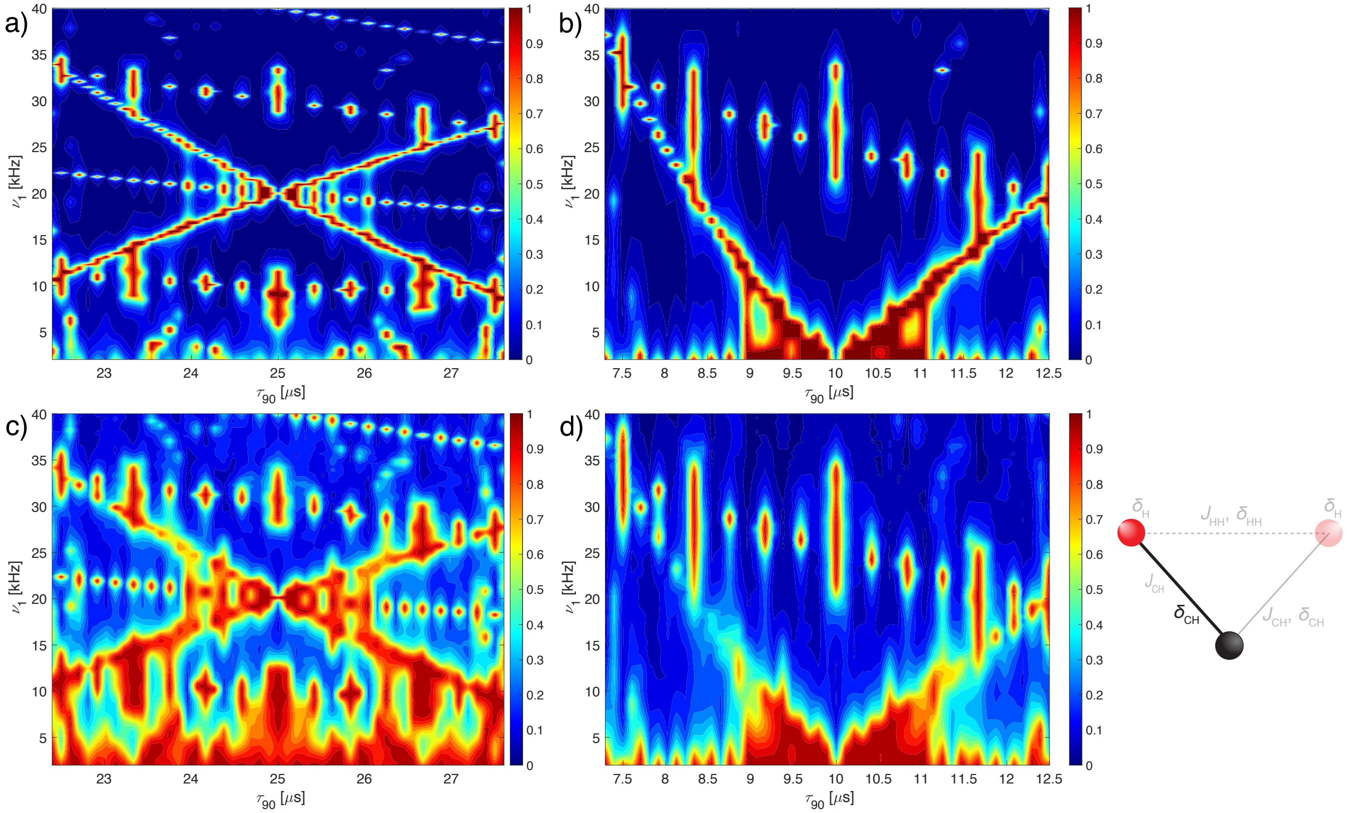

Figure S6. Plot of the line intensities in a three-spin CH_2 system as a function of the pulse length τ_{90} and the rf-field amplitude ν_1 for the WALTZ-16 pulse sequence assuming ideal rectangular pulses around the conditions $\omega_r/\omega_1 = 10$ (left side) and $\omega_r/\omega_1 = 4$ (right side). The topmost row shows the analytical calculations of the resonance intensity a) around $k_0 = 960$, ν_1 = 10 kHz, τ_{90} = 25 µs, b) around $k_0 = 384$, ν_1 = 25 kHz, τ_{90} = 10 µs. The residual coupling was converted into a Gaussian line with the corresponding line width and the line intensity of this line is plotted to make the plots easier comparable to the numerical simulations. The second row shows the line height of the numerical simulations for a two-spin system with only a heteronuclear dipolar coupling of $\delta_{CH}/(2\pi)$=-45.3 kHz in c) around ν_1 = 10 kHz, τ_{90} = 25 µs, in d) around ν_1 = 25 kHz, τ_{90} = 10 µs.

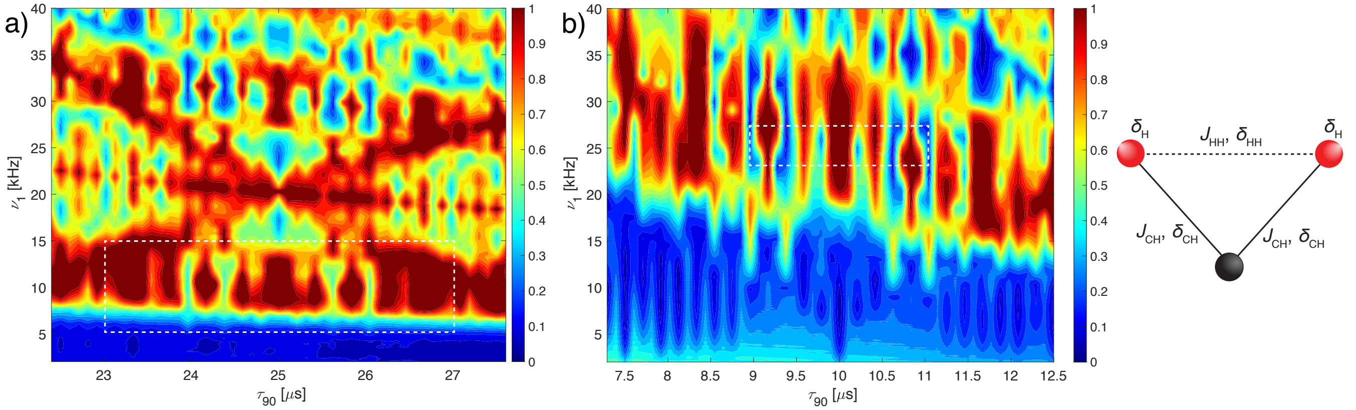

Figure S7. Plot of the line intensities as a function of the pulse length τ_{90} and the rf-field amplitude ν_1 for the WALTZ-16 pulse sequence assuming ideal rectangular pulses around the conditions $\omega_r/\omega_1 = 10$ (left side) and $\omega_r/\omega_1 = 4$ (right side). The line height of the numerical simulations with all interactions is shown in a) around ν_1 = 10 kHz, τ_{90} = 25 µs, in b) around ν_1 = 25 kHz, τ_{90} = 10 µs. The parameters used for the simulations can be found in the SI section S1. The white dashed box indicates the area which is covered by the experimental data in Fig. S8

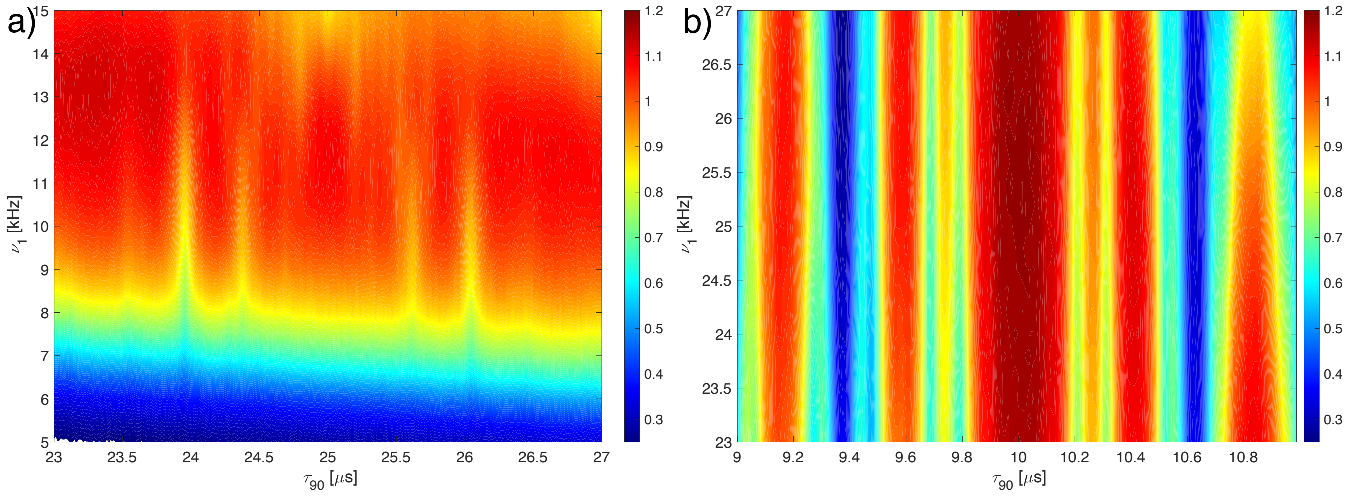

Figure S8. Plot of the experimentally measured line intensities as a function of the pulse length τ_{90} and the rf-field amplitude ν_1 for the WALTZ-16 pulse sequence in glycine ethyl ester around the conditions $\omega_r/\omega_1 = 10$ (left side) and $\omega_r/\omega_1 = 4$ (right side). The line height of the CH_2 group in a) around ν_1 = 10 kHz, τ_{90} = 25 µs, in b) around ν_1 = 25 kHz, τ_{90} = 10 µs.

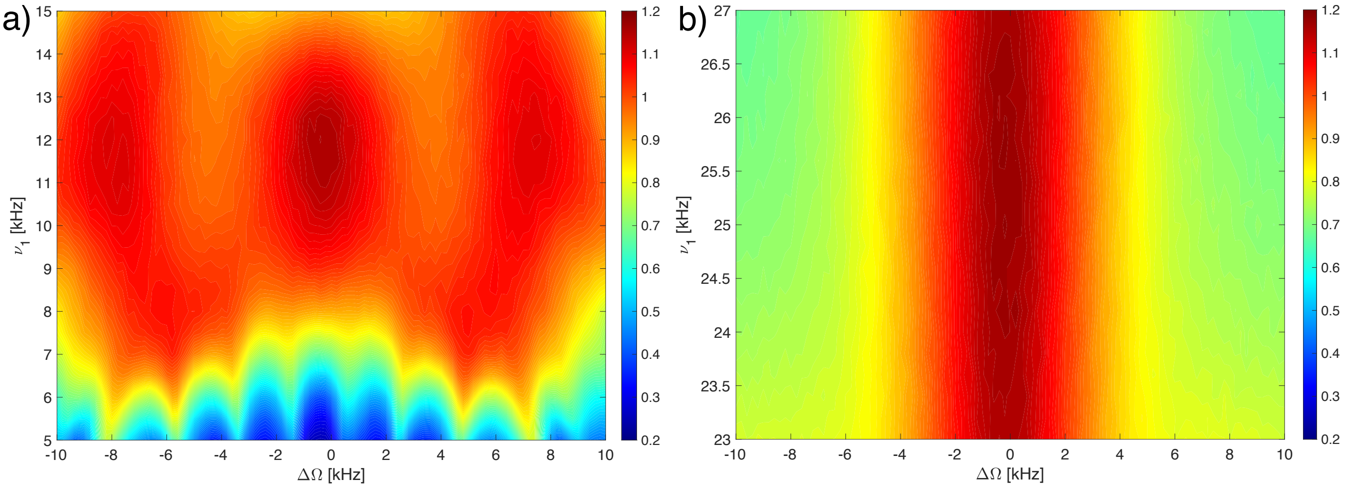

Figure S9. Plot of the experimentally measured line intensities as a function of the irradiation offset on the protons ($\Delta\Omega$) and the rf-field amplitude ν_1 for the WALTZ-16 pulse sequence in glycine ethyl ester around the conditions a) $\omega_r/\omega_1 = 10$ corresponding to τ_{90} = 25 µs around ν_1 = 10 kHz and b) $\omega_r/\omega_1 = 4$ corresponding to τ_{90} = 10 µs around ν_1 = 25 kHz.

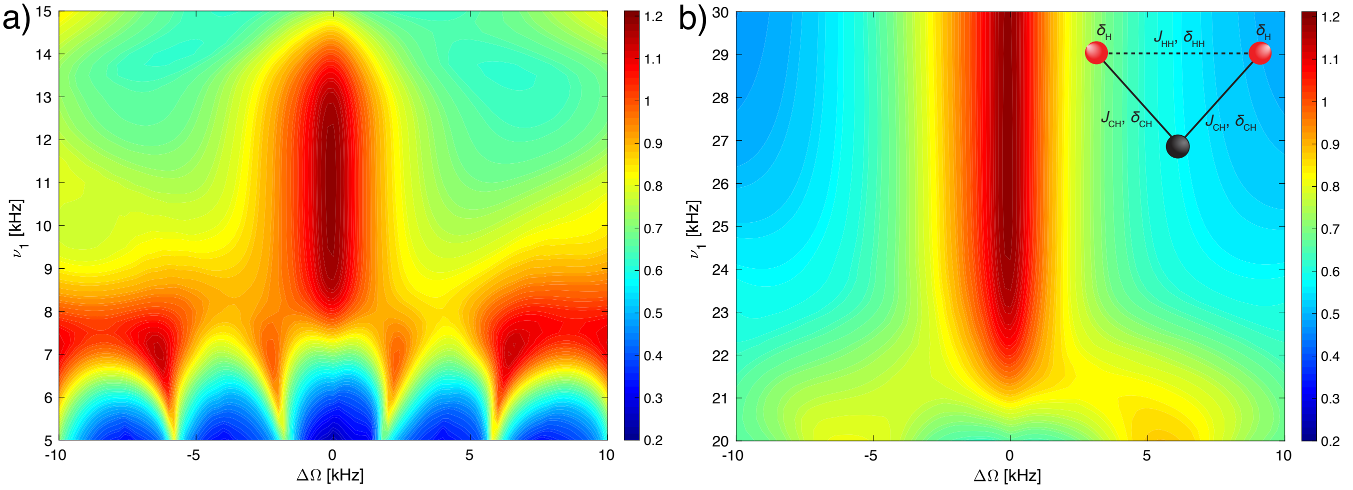

Figure S10. Plot of the numerically simulated line intensities as a function of the irradiation offset on the protons ($\Delta\Omega$) and the rf-field amplitude ν_1 for the WALTZ-16 pulse sequence in three-spin CH_2 system around the conditions a) $\omega_r/\omega_1 = 10$ corresponding to τ_{90} = 25 µs around ν_1 = 10 kHz and b) $\omega_r/\omega_1 = 4$ corresponding to τ_{90} = 10 µs around ν_1 = 25 kHz.

S3 Additional figures

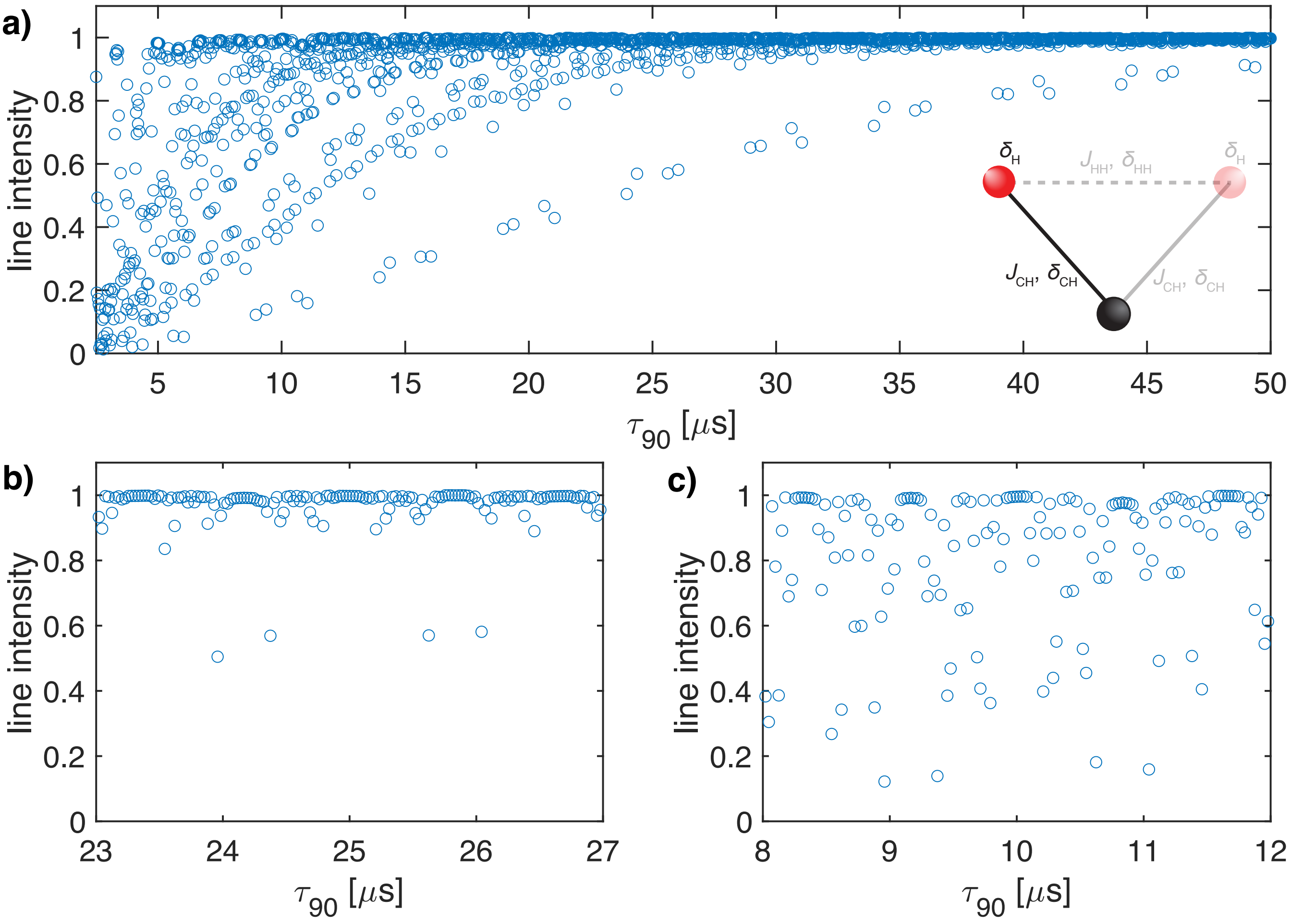

Figure S11. Plot of the simulated line intensity in a two-spin system with the heteronuclear dipolar coupling and the I-spin CSA tensor as a function of the pulse length τ_{90} for the WALTZ-64 pulse sequence assuming ideal rectangular pulses. The corresponding rf-field amplitude for each value of τ_{90} can be calculated as $\nu_1 = 1/(4\tau_{90})$. The pulse length correspond to values of $\tau_{90} = k_0\tau_r/(4 * z_0)$. a) Complete range from τ_{90} = 2.5 to 50 µs corresponding to k_0 = 96 to 1920. b) Enlarged range around τ_{90} = 25 µs corresponding to an ideal B_1 field of 10 kHz. c) Enlarged range around τ_{90} = 10 µs corresponding to an ideal B_1 field of 25 kHz.

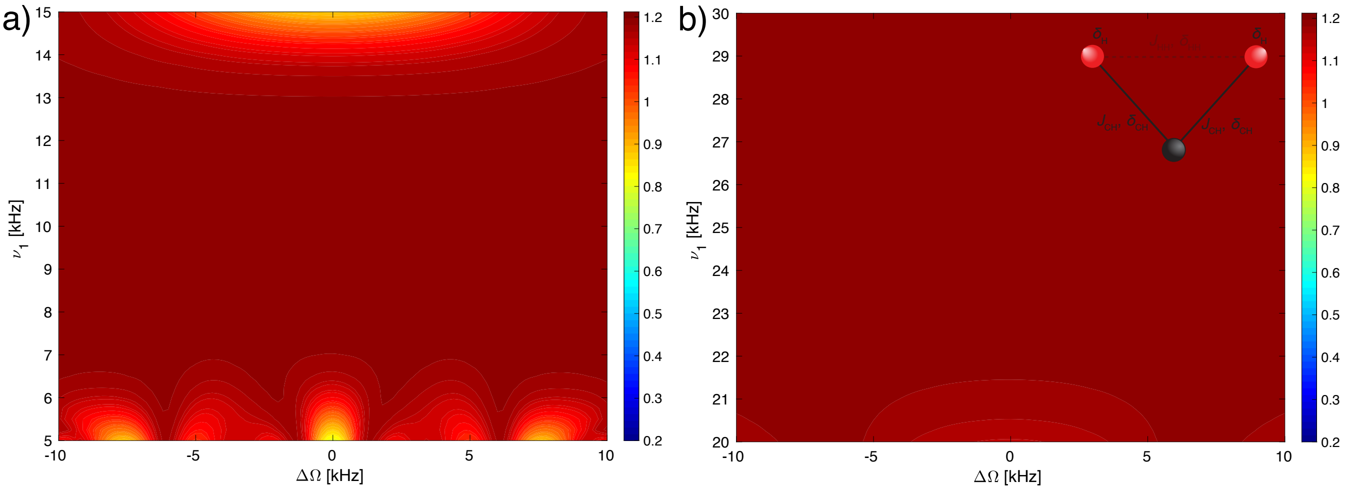

Figure S12. Plot of the numerically simulated line intensities as a function of the irradiation offset on the protons ($\Delta\Omega$) and the rf-field amplitude ν_1 for the WALTZ-64 pulse sequence in three-spin CH_2 system without a homonuclear dipolar coupling around the conditions a) $\omega_r/\omega_1 = 10$ corresponding to τ_{90} = 25 µs around ν_1 = 10 kHz and b) $\omega_r/\omega_1 = 4$ corresponding to τ_{90} = 10 µs around ν_1 = 25 kHz.

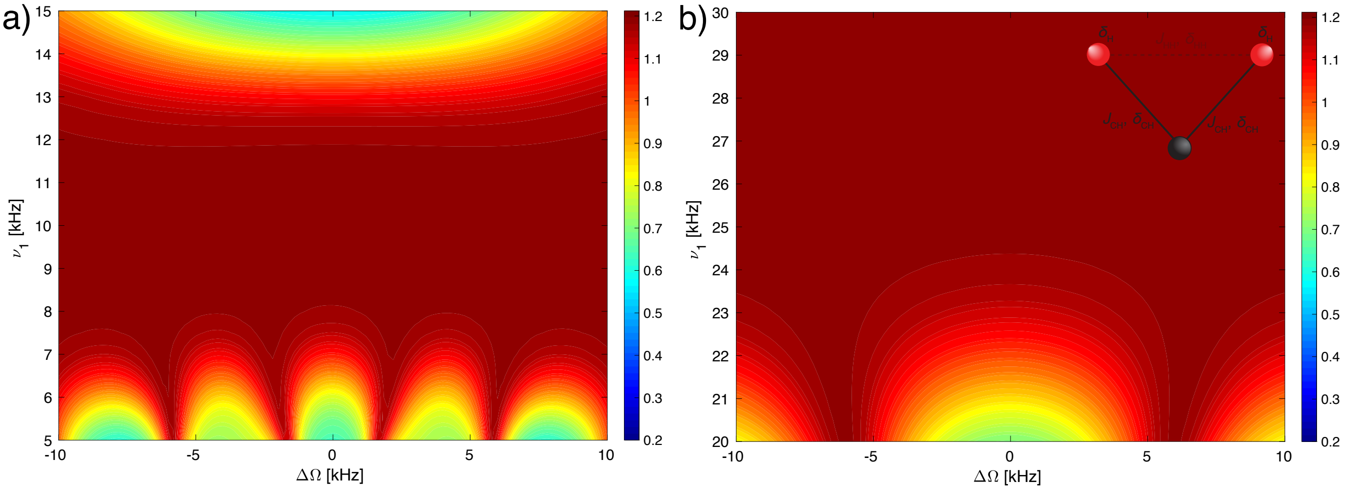

Figure S13. Plot of the numerically simulated line intensities as a function of the irradiation offset on the protons ($\Delta\Omega$) and the rf-field amplitude ν_1 for the WALTZ-16 pulse sequence in three-spin CH_2 system without a homonuclear dipolar coupling around the conditions a) $\omega_r/\omega_1 = 10$ corresponding to τ_{90} = 25 µs around ν_1 = 10 kHz and b) $\omega_r/\omega_1 = 4$ corresponding to τ_{90} = 10 µs around ν_1 = 25 kHz.

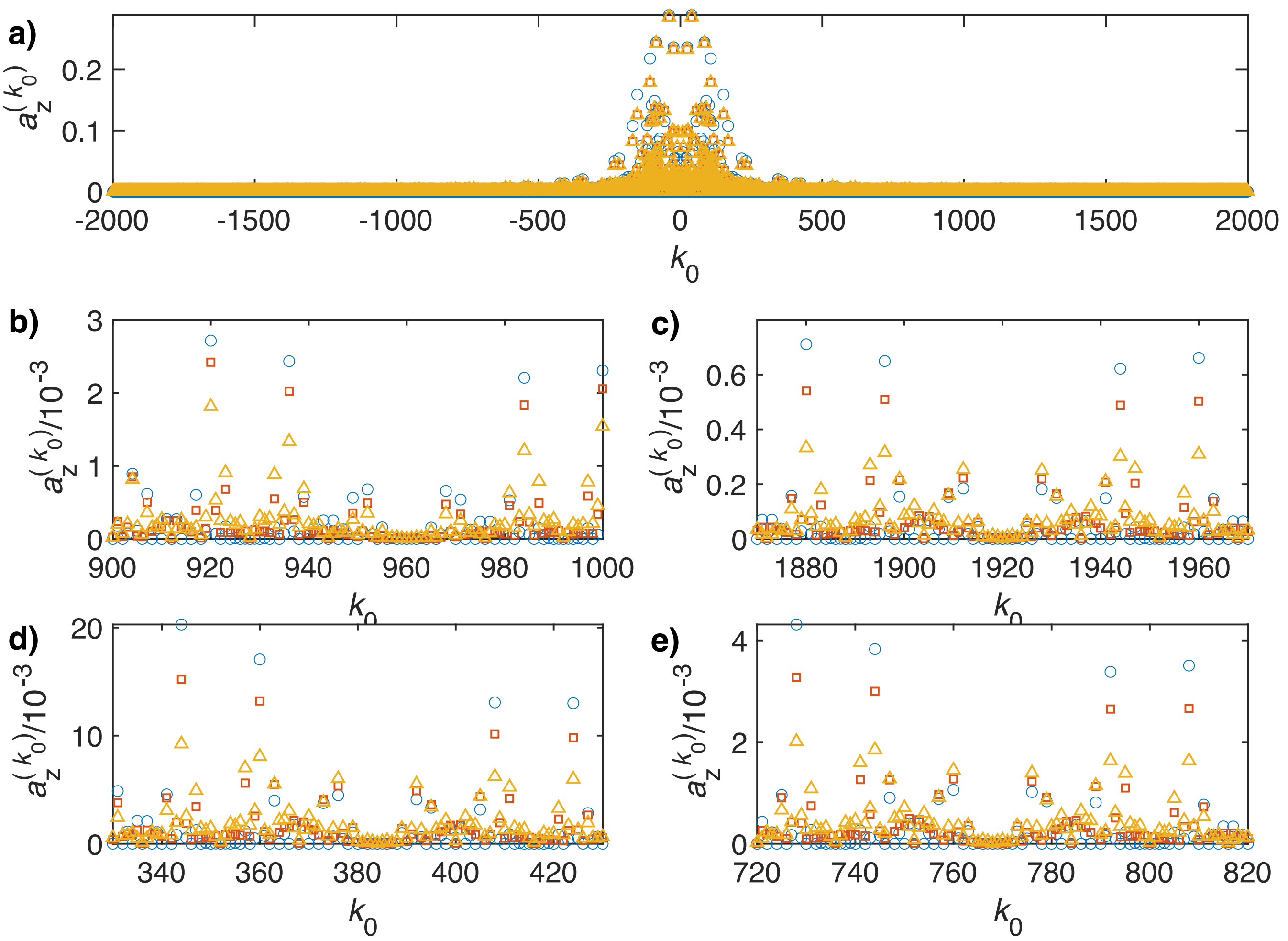

Figure S14. Plot of the Fourier coefficients $a_z^{(k_0)}$ as a function of k_0 for the WALTZ-64 pulse sequence assuming ideal rectangular pulses. Blue circles correspond to the ideal B_1 field (same data as Fig. 1), red squares to a B_1 field that is decreased by 10% and yellow triangles to one that is decreased by 20%. a) Complete range from $k_0 = -2000$ to 2000. b) Enlarged range around $k_0 = 960$ corresponding to a ratio of $\omega_\mathrm{r}/\omega_1 = 10$ for $n_0 = 1$. c) Enlarged range around $k_0 = 1920$ corresponding to a ratio of $\omega_\mathrm{r}/\omega_1 = 10$ for $n_0 = 2$. d) Enlarged range around $k_0 = 384$ corresponding to a ratio of $\omega_\mathrm{r}/\omega_1 = 4$ for $n_0 = 1$. c) Enlarged range around $k_0 = 768$ corresponding to a ratio of $\omega_\mathrm{r}/\omega_1 = 4$ for $n_0 = 2$.

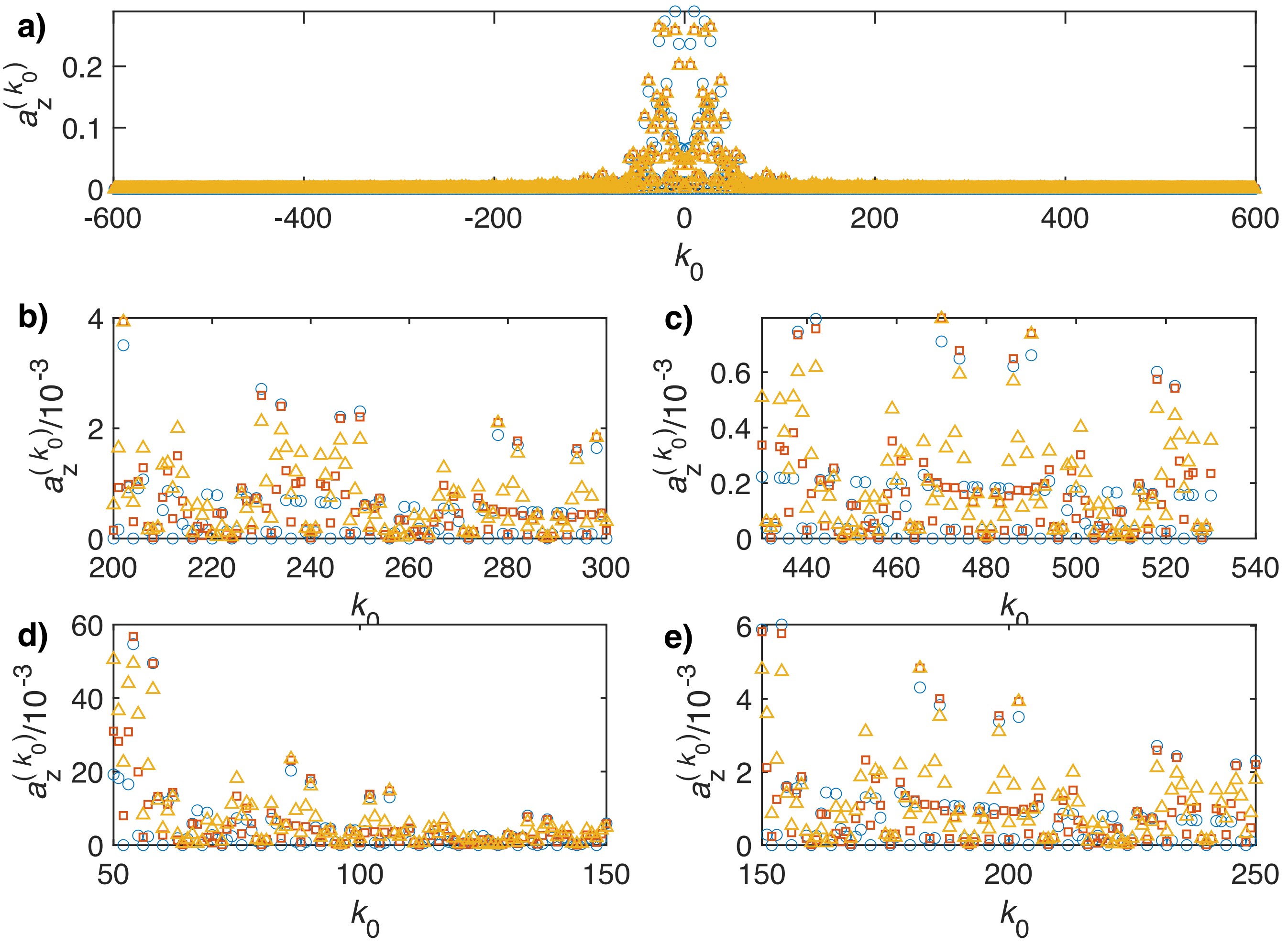

Figure S15. Plot of the Fourier coefficients $a_z^{(k_0)}$ as a function of k_0 for the WALTZ-16 pulse sequence assuming ideal rectangular pulses. Blue circles correspond to the ideal B_1 field (same data as Fig. S1), red squares to a B_1 field that is decreased by 10% and yellow triangles to one that is decreased by 20%. a) Complete range from $k_0 = -600$ to 600. b) Enlarged range around $k_0 = 240$ corresponding to a ratio of $\omega_\mathrm{r}/\omega_1 = 10$ for $n_0 = 1$. c) Enlarged range around $k_0 = 480$ corresponding to a ratio of $\omega_\mathrm{r}/\omega_1 = 10$ for $n_0 = 2$. d) Enlarged range around $k_0 = 96$ corresponding to a ratio of $\omega_\mathrm{r}/\omega_1 = 4$ for $n_0 = 1$. c) Enlarged range around $k_0 = 192$ corresponding to a ratio of $\omega_\mathrm{r}/\omega_1 = 4$ for $n_0 = 2$.

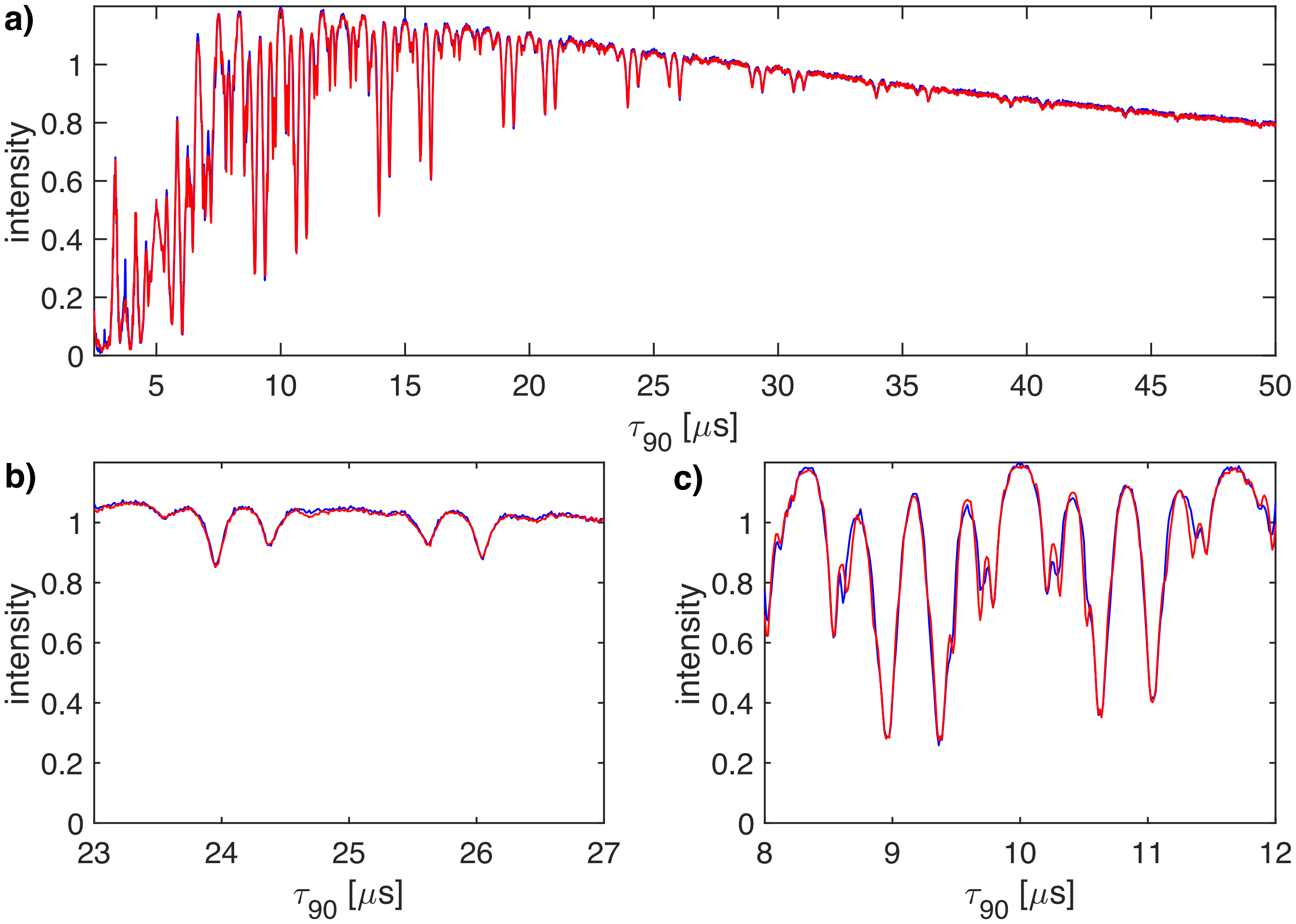

Figure S16. Plot of the measured line intensity of the CH_2 group in 1,2-^{13}C glycine ethyl ester as a function of the pulse length τ_{90} for the WALTZ-16 (red) and the WALTZ-64 (blue) pulse sequence at 100 kHz MAS. The rf-field amplitude was adjusted such that the flip angle was always 90°. The increment of the pulse length was set to 12.5 ns. a) Complete range from τ_{90} = 2.5 to 50 µs. b) Enlarged range around τ_{90} = 25 µs corresponding to a B_1 field around 10 kHz. c) Enlarged range around τ_{90} = 10 µs corresponding to a B_1 field around 25 kHz. While the numerical calculations and simulations of Figs. S1-S3 could be carried out exactly on the resonance conditions, this was not possible for the experimental data due to the limited time resolution of the pulse programmer and the limited stability of the MAS rotation. Therefore, the experimental data are displayed as a line plot with the highest possible time resolution.
